# Efficacy of belimumab on extra-renal lupus in patients with lupus nephritis and end-stage renal disease

**DOI:** 10.3389/fmed.2025.1717280

**Published:** 2026-01-05

**Authors:** Jia-Qi Teng, Wen-Ping Guo, Jing Xu, Meng Tan, Ying Tan, Zhen Qu, Hai-Hua Su, Feng Yu

**Affiliations:** 1 Division of Renal, Department of Medicine, Peking University International Hospital, Beijing, China; 2 Division of Renal, Department of Medicine, Tianjin PKU Care CNOOC Hospital, Tian Jin, China; 3 Department of Obstetrics and Gynecology, Peking University International Hospital, Beijing, China; 4 Division of Renal, Department of Medicine, Key Laboratory of CKD Prevention and Treatment, Key Laboratory of Renal Disease, Ministry of Health of China, Ministry of Education of China, Peking University First Hospital, Institute of Nephrology, Peking University, Beijing, China

**Keywords:** belimumab, end-stage renal disease, evaluation, lupus nephritis, safety

## Abstract

**Background:**

Lupus nephritis (LN) is a severe complication of systemic lupus erythematosus (SLE), with approximately 20–30% of LN patients progressing to end-stage renal disease (ESRD) within a decade. Persistent extra-renal SLE activity, cardiovascular events, and infections significantly contribute to adverse outcomes. Current therapeutic strategies primarily rely on glucocorticoids and immunosuppressants; however, the adverse effects of these agents are intensified in ESRD patients. Belimumab, a B-lymphocyte-targeted biologic agent, has demonstrated efficacy in managing SLE but lacks sufficient data regarding its application in ESRD-LN patients. This study aims to evaluate the role of belimumab in controlling extra-renal disease activity, optimizing medication use, and assessing safety in ESRD-LN patients.

**Methods:**

This retrospective analysis involved eight LN patients with ESRD (eGFR <15 mL/min/1.73 m^2^ or on hemodialysis) who received belimumab treatment. Data on SLE manifestations, medications, and laboratory parameters were collected before and after ESRD onset. The primary endpoints included changes in extra-renal Systemic Lupus Erythematosus Disease Activity Index (SLEDAI) scores, adjustments in prednisone dosages, and the incidence of infection episodes during belimumab therapy.

**Results:**

This study analyzed data from eight ESRD-LN patients treated with belimumab over a median follow-up period of 28.01 ± 14.57 months. Belimumab significantly reduced extra-renal SLEDAI scores (from 6.13 ± 3.09 to 1.50 ± 1.41, *p* < 0.01) and improved complement C3 levels (from 0.64 ± 0.12 g/L to 0.8 ± 0.16 g/L). Glucocorticoid dosages were markedly reduced (from 21.25 ± 9.64 mg/day to 3.91 ± 3.98 mg/day), with three patients discontinuing glucocorticoid therapy entirely. Mild infections were observed, but no severe infections requiring mechanical ventilation were reported.

**Conclusion:**

Belimumab appears to be a promising therapeutic option for ESRD-LN patients, effectively controlling extra-renal disease activity, reducing glucocorticoid use, and demonstrating a favorable safety profile. Further research is required to optimize dosing strategies and to validate the long-term efficacy and safety of belimumab in this high-risk population.

## Introduction

Systemic lupus erythematosus (SLE) is a chronic, inflammatory autoimmune disorder characterized by diverse clinical manifestations. Lupus nephritis (LN) refers to kidney injury caused by SLE resulting from renal damage induced by circulating or *in situ* immune complex deposition. In Western countries, the occurrence of LN ranges from 30 to 50%, whereas higher rates (50–70%) are reported among SLE patients in Asia and Latin America (1). Approximately 20 to 30% of LN patients progress to end-stage renal disease (ESRD) within a decade (2).

A study on post-ESRD survival in LN patients reported 5-year and 10-year survival rates of 70–80% and 50–60%, respectively, which were significantly lower than those observed in general ESRD patients (85–90% 5-year survival) (3). Reduced survival is associated with the significant risk of cardiovascular disease (CVD), infections (particularly urinary tract and pulmonary infections), and persistent extra-renal SLE activity. Approximately 43% of SLE patients progressing to ESRD exhibit ongoing extra-renal manifestations (e.g., arthritis, rashes, central nervous system involvement, and alopecia), with 44% continuing to display these symptoms post-dialysis (4, 5). Sustained immune activity further increases the risks of vasculitis, atherosclerosis, and CVD events, contributing to poor outcomes (6). Active management remains essential in this population. The current clinical approach still relies on glucocorticoids and conventional immunosuppressants, though their adverse effects (e.g., infections and metabolic complications) are amplified in ESRD patients due to unique physiological vulnerabilities. KDIGO 2024 Clinical Practice Guideline for the management of lupus nephritis (the 2024 KDIGO guidelines) recommends renal replacement therapies (hemodialysis and peritoneal dialysis) or transplantation for LN patients with kidney failure (7). However, no evidence-based recommendations exist for glucocorticoids, immunosuppressants, or biologics in this population, highlighting the critical need for large-scale clinical studies to establish therapeutic protocols.

Belimumab is a B-lymphocyte-targeted biologic agent that prevents the activity of B-lymphocyte stimulator (BLyS), blocks B-cell proliferation and differentiation, and induces B-cell exhaustion and apoptosis, thereby reducing autoantibody production and attenuating autoimmune responses. The efficacy and safety of SLE treatment have been evaluated in several clinical trials (8, 9). However, none of the aforementioned clinical trials included patients with an estimated glomerular filtration rate (eGFR) < 30 mL/min. There remains a lack of research into the therapeutic management of this patient population. Only a few reports have reported that belimumab may reduce disease activity and facilitate dialysis discontinuation in lupus nephritis patients requiring renal replacement therapy (RRT) (10–12). In patients undergoing hemodialysis (HD) or peritoneal dialysis (PD), belimumab has been associated with improvements in extra-renal disease activity and a favorable safety profile (13, 14), although the current evidence is largely limited to case reports. This retrospective study enrolled LN patients who had progressed to ESRD and received belimumab therapy, aiming to evaluate its role in controlling extra-renal disease activity, optimizing concomitant medication strategy, and evaluating long-term safety profiles in this population.

## Methods

This multicenter retrospective analysis included patients with ESRD diagnosed with LN at Peking University International Hospital and Peking University First Hospital between 2020 and 2024. The inclusion criteria were age >18 years; fulfillment of the 1997 American College of Rheumatology (ACR) classification criteria for SLE; renal pathology consistent with the 2018 revised International Society of Nephrology/Renal Pathology Society (ISN/RPS) classification for LN; or the following clinical features: (1) persistent proteinuria >0.5 g/24 h, or urine protein-to-creatinine ratio (uPCR) > 500 mg/g (50 mg/mmol); (2) active urinary sediment [excluding urinary tract infection, defined as urinary leukocytes >5/high power field (HPF), urinary erythrocytes >5/HPF], or red blood cell casts, or white blood cell casts; (3) unexplained decline in eGFR; (4) meeting criteria for chronic kidney disease (CKD) stage 5 (eGFR <15 mL/min/1.73 m^2^) or undergoing hemodialysis (HD); and (5) absence of other secondary diseases causing ESRD. Data on SLE-related clinical manifestations, medication regimens, and laboratory parameters were collected before and after the onset of end-stage renal disease. SLE disease manifestations and laboratory parameters were extracted from clinical records. Definitions included: arthritis (≥2 painful joints with signs of inflammation), lupus-associated rash, alopecia, oral ulcers, serositis (pericardial pain accompanied by at least one of the following: friction rub, effusion, or confirmation via ECG/echocardiography), central nervous system (CNS) manifestations documented seizures (after rigorous medical record review and exclusion of uremic, metabolic, or drug-related etiologies by neurology/nephrology evaluation) and/or psychosis; complement levels [CH50, C3, or C4 below the lower limit of normal (LLN)], anti-dsDNA antibodies [titers exceeding the upper limit of normal (ULN)], leukopenia (<3,000 white blood cells/mm^3^), and thrombocytopenia (<100,000 platelets/mm^3^). Therapeutic regimens included prednisone, azathioprine, cyclophosphamide, mycophenolate mofetil, tacrolimus, belimumab (BEL), and rituximab (RTX). All clinical and biological data were systematically extracted from electronic medical records. Key endpoints assessed were (1) changes in extra-renal SLE Disease Activity Index (SLEDAI) scores, (2) prednisone dosage adjustments, and (3) incident infection episodes during belimumab treatment.

Infections were graded according to the Common Terminology Criteria for Adverse Events (CTCAE) version 5.0 (21), with grades 3–5 classified as serious infections.

The follow-up period began at the time of belimumab initiation and extended until the study end date (31 December 2024), or 3 months after belimumab discontinuation, or the date of kidney transplantation, whichever occurs first.

This study was approved by the ethics committee of Peking University International Hospital and Peking University First Hospital, and was conducted with informed consent from each patient. The ethics approval number is Peking University First Hospital (No. 2017 [1333]).

### Statistical methods

All analyses were performed using SPSS 27. Continuous data were expressed as mean and standard deviation or median and interquartile range (IQR), as appropriate. The Kruskal–Wallis H (skewed distribution) test was used to determine any statistical differences in the means and proportions before and after treatment with belimumab. A *p*-value of <0.05 was considered statistically significant.

## Results

### Baseline demographic and clinical characteristics

**Figure 1:**
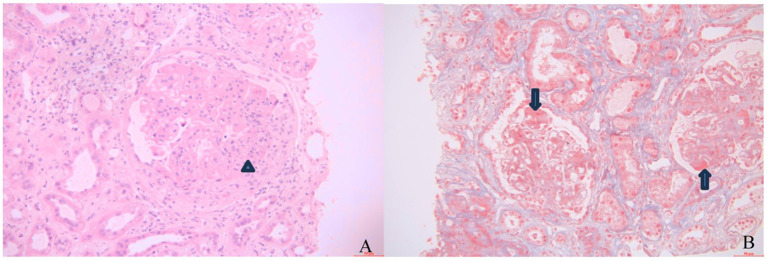
Renal biopsy exhibits moderate-to-severe diffuse hyperplasia of mesangial cells and matrix, with segmental hypercellularity in capillary lumina accompanied by a small number of neutrophil infiltrates (**A**, triangle; Hematoxylin and Eosi0n staining, ×200), diffuse thickening of the basement membrane, spike and chain-loop formation, pseudodouble-track formation, and subepithelial, intramembranous, and mesangial eosinophilic deposits (**B**, arrow; Periodic acid-Schiff stain, ×200). The pathological diagnosis was focal proliferative lupus nephritis with membranous lupus nephritis and thrombotic microangiopathy (TMA) renal injury (Class IV + V), AI = 6, CI = 7, activity index (AI): endocapillary hypercellularity 1, neutrophil infiltration 1, fibrinoid necrosis 0, cellular/fibrocellular crescents 1*2, wire loops/microthrombi 0, interstitial inflammatory cell infiltration 2; and chronicity index (CI): glomerulosclerosis 2, fibrous crescents 0, tubular atrophy 3, and interstitial fibrosis 2.

This study retrospectively analyzed eight LN patients who had progressed to end-stage renal disease and were maintained on belimumab treatment. Among them, seven patients had been regularly undergoing maintenance hemodialysis treatment, and one patient had not received renal replacement therapy (mean follow-up: 28.01 ± 14.57 months). Key demographic and clinical characteristics: The cohort comprised three male and five female patients, aged 32–58 years, with SLE duration ranging from 2 to 33 years. Time on dialysis varied from 3 months to 13 years. Six patients had biopsy-proven lupus nephritis (LN), including five with class IV LN and one with combined class III + V LN with thrombotic microangiopathy (TMA) (Figure 1). Previous immunosuppressive regimens included glucocorticoids, cyclophosphamide, mycophenolate mofetil, azathioprine, cyclosporine, tacrolimus, and leflunomide. Three patients have received adjunctive rituximab therapy. Clinical characteristics of patients prior to belimumab initiation are summarized in Table 1, and their baseline data plus the belimumab administration regimen at treatment onset are presented in Table 2.

**Table 1:** Clinical characteristics of the study population before belimumab initiation.

Patient	Age, Sex	Disease duration of SLE (years)	Disease duration of LN (years)	Duration of dialysis	LN class	Any history of extra-renal SLE manifestations	Antibodies, complement levels	Peak extra-renal SLEDAI score	Extra-renal SLICC damage index	Previous therapies
1	55, F	33	33	11 years	IV	Pancytopenia, arthralgia, fever	ANA+, dsDNA+, C3↓	10	1 (Osteoporosis with fracture)	GC, CYC, MMF, AZA, CSA
2	58, F	23	15	13 years	IV	Rash, pancytopenia	ANA+, dsDNA+, C3↓, Anti-nucleosome +, Anti-histone +, SSA+, SSB+, Anti-*β*2GPI+	8	0	GC, CYC, MMF, *Tripterygium wilfordii*, HCQ
3	51, F	20	6	Pre-dialysis	IV	Rash, arthralgia	ANA+, C3↓C4↓, LA+, Anti-β2GPI+	10	2 (retinal change, premature gonadal failure)	GS, MMF
4	32, F	12	12	7 months	III + V + TMA	Alopecia, rash, arthralgia, serositis, TMA	ANA+, dsDNA+, C3↓	14	0	GC, LF, CSA, MMF, CYC, HCQ, RTX
5	29, M	13	13	Pre-dialysis	IV	Fever	ANA+, dsDNA+, C3↓, C4↓, U1-RNP+, Sm-D1 +, LA+	5	0	GC, CYC, MMF, LF, RTX
6	35, F	2	2	1 year	ND	APS with pulmonary embolism, serositis, TMA	ANA+, C3↓, dsDNA+, aCL+	8	1 (pulmonary infarction)	GC, CYC, CsA, HCQ, sirolimus, MMF
7	34, M	3	3	2 years	IV	Rash, thrombocytopenia	ANA+, dsDNA+ C3↓	7	0	GC, CYC, RTX
8	30, M	6	6	3 months	ND	APS with pulmonary embolism, rash, serositis, TMA	C3↓, C4↓, dsDNA+, LA+	7	1 (diabetes)	GC, CSA, Tacrolimus, MMF, RTX, HCQ

F, female; M, male; TMA, thrombotic microangiopathy; ND, not done; APS, antiphospholipid syndrome; anti-β2GPI, anti-β₂-glycoprotein I; LA, lupus anticoagulant; aCL, anticardiolipin; GC, glucocorticoid; CYC, cyclophosphamide; MMF, mycophenolate mofetil; AZA, azathioprine; CsA, cyclosporine A; HCQ, hydroxychloroquine; RTX, rituximab.

**Table 2:** Baseline clinical characteristics and belimumab treatment details.

Patient	Extra-renal SLEDAI score at belimumab initiation	Concomitant medications at baseline	GC dosage at baseline^#^ (mg/day)	Primary indication for belimumab	Belimumab dose (mg/kg)	Dosing frequency
1	9 (dsDNA+, C3↓, C4↓, arthritis, thrombocytopenia)	GC	20	Control of extra-renal lupus activity	4.3 → 6.4	Q4W → Q8W
2	10 (dsDNA+, C3↓and C4↓, rash, arthritis)	GC, HCQ	20	Control of extra-renal lupus activity	6.5	Q4W → Q8W
3	4 (C3↓, rash)	GC	15	Steroid-sparing strategy	6.8	Q4W
4	2 (C3↓, C4↓)	GC, RTX, HCQ	12.5	Relapse prevention (listed for kidney transplantation)	6.2	Q4W
5	2 (C3↓)	GC, RTX	30	Steroid-sparing strategy	10	Q4W → Q8W
6	8 (dsDNA+, C3↓, thrombocytopenia, serositis)	GC, MMF, sirolimus	30	Control of extra-renal lupus activity	8	Q4W
7	5 (dsDNA+, C3↓, thrombocytopenia)	GC, CYC	7.5	Relapse prevention (listed for kidney transplantation)	6.9	Q4W
8	5 (dsDNA+, C3↓and C4↓, leukopenia)	GC, HCQ, RTX	35	Steroid-sparing strategy	3.6 → 7.9	Q4W

GC, glucocorticoid; HCQ, hydroxychloroquine; RTX, rituximab; MMF, mycophenolate mofetil; CYC, cyclophosphamide; Q4W, every 4 weeks; Q8W, every 8 weeks. ^#^prednisone-equivalent.

### Dynamics of extra-renal disease activity parameters

**Figure 2:**
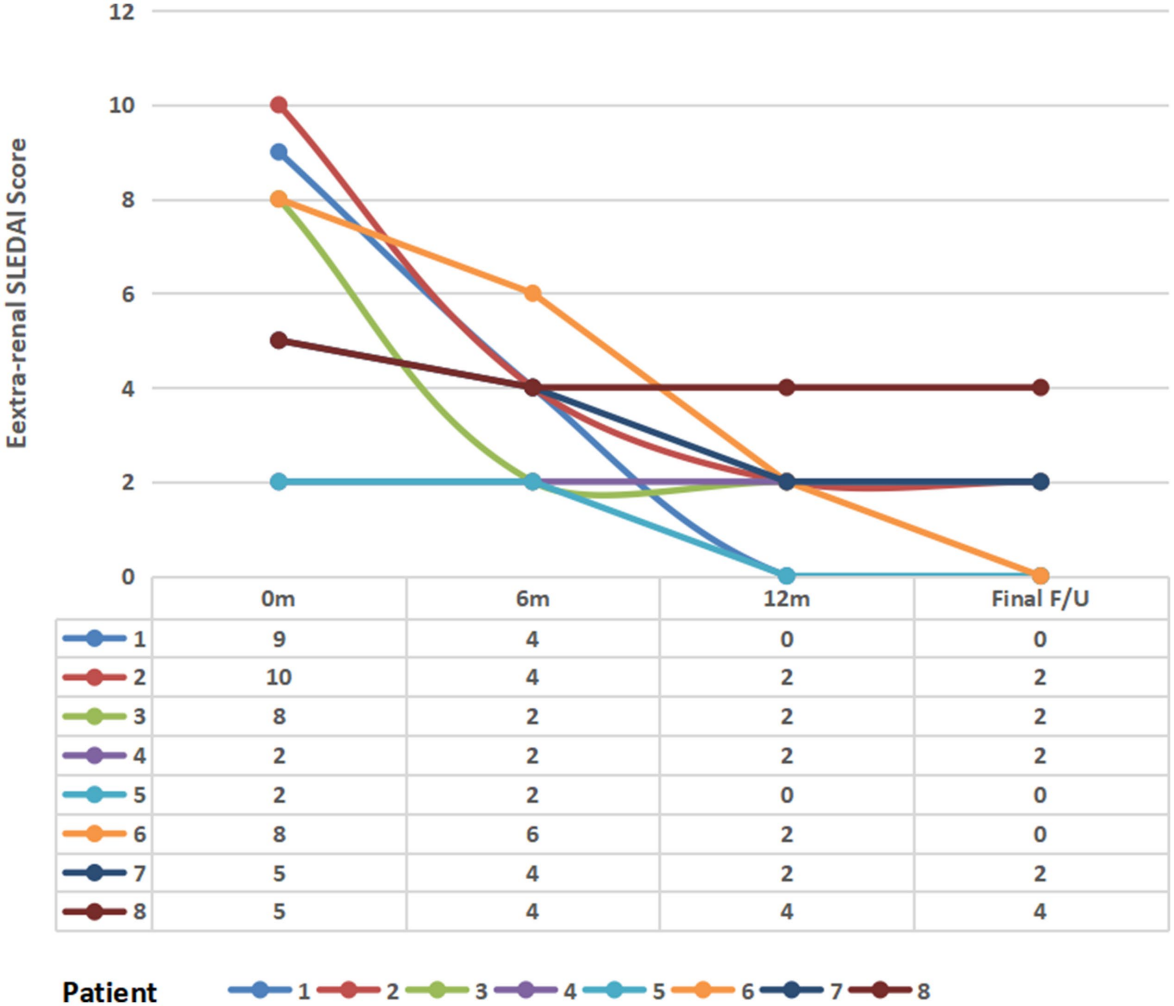
Longitudinal changes in the extra-renal SLEDAI score following belimumab therapy. m, month(s); final F/U, final follow-up visit.

The patient’s prior extra-renal involvement primarily affected the hematological system, mucocutaneous tissues, and arthritis. Serological profiles at baseline: All patients had elevated anti-dsDNA antibody levels above the institutional upper limits of normal. The mean complement levels are C3 (0.64 ± 0.12 g/L) and C4 (0.15 ± 0.09 g/L). The mean extra-renal SLEDAI score was 6.13 ± 3.09. All patients received weight-based belimumab therapy (mean dose: 6.54 ± 2.0 mg/kg), and changes in disease activity and complement levels were assessed at 3, 6, and 12 months. During belimumab treatment, extra-renal disease activity was assessed at 3, 6, and 12 months as well as the final follow-up visit. Results demonstrated a significant downward trend in mean extra-renal SLEDAI scores over time: 6.13 ± 3.09 at baseline, 3.50 ± 1.41 at 6 months, 1.75 ± 1.28 at 12 months, and 1.50 ± 1.41 at final follow-up (*p* < 0.01 for trend) (Figure 2). Mean complement C3 levels demonstrated an overall upward trend: 0.76 ± 0.16 g/L at 3 months, 0.85 ± 0.22 g/L at 6 months, 0.76 ± 0.19 g/L at 12 months, and 0.8 ± 0.16 g/L at final follow-up (Supplementary Table 1 for details).

### Medication profiles

**Figure 3:**
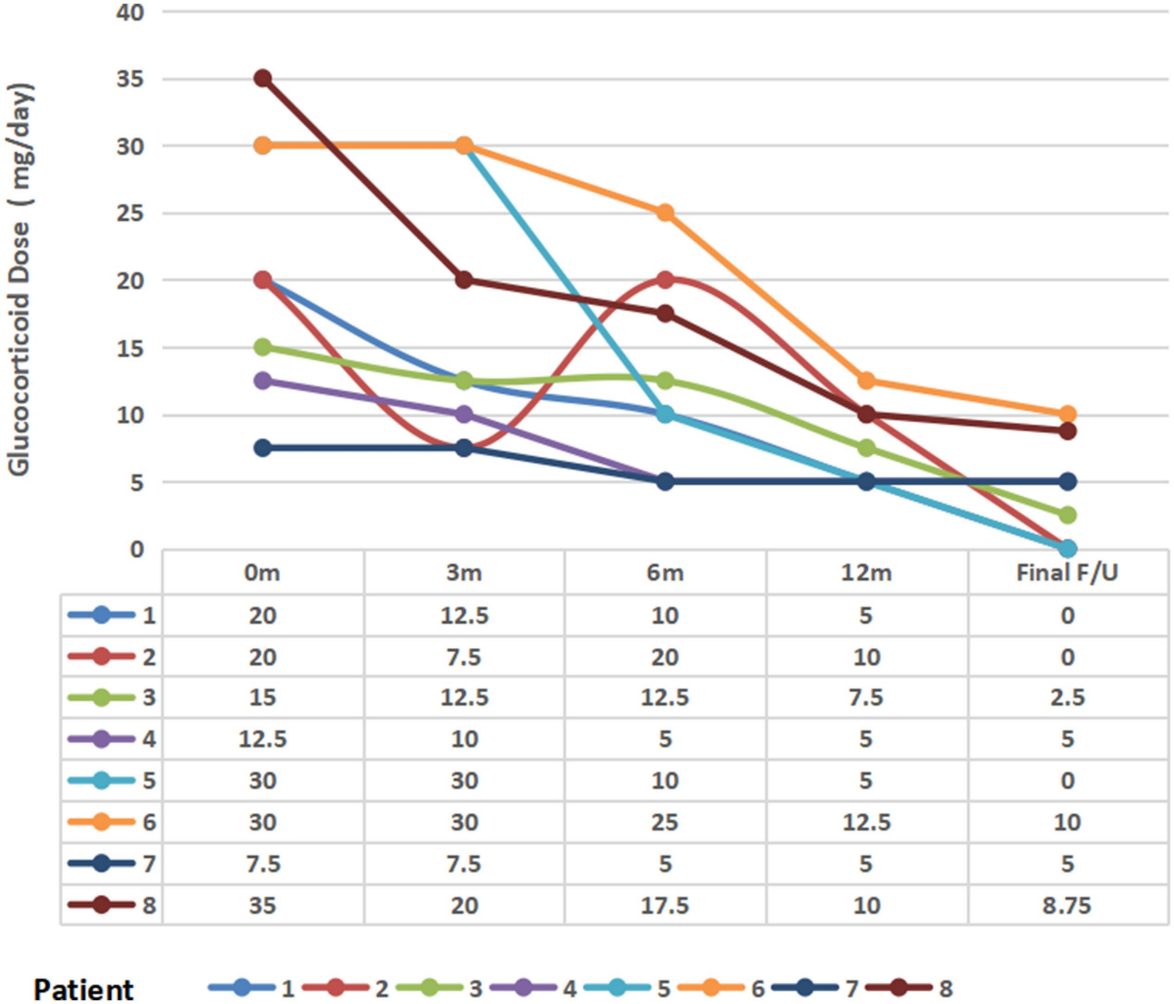
Change in glucocorticoid dose (prednisone-equivalent) following belimumab therapy. m, Month(s); final F/U, final follow-up visit.

All eight patients received glucocorticoid-containing regimens. The baseline prednisone dosage was 21.3 ± 9.6 mg/day, which progressively decreased to 16.25 ± 9.35 mg/day at 3 months, 13.13 ± 7.17 mg/day at 6 months, 7.50 ± 2.99 mg/day at 12 months, and 3.91 ± 3.98 mg/day at final follow-up during belimumab treatment. Three patients (37.5%) successfully discontinued glucocorticoids (Figure 3).

### Treatment-related infections and adverse events

Among the eight patients receiving belimumab, two discontinued treatment—one due to recurrent pulmonary infections (*n* = 2) with concomitant leukopenia (< 3.0 × 10^9^/L), and another following renal transplantation due to stable extra-renal disease activity. The remaining six patients continued therapy through final follow-up, experiencing mild infections, including one case of periodontal abscess, four COVID-19 infections (two with secondary pneumonia), and one cuffed catheter tunnel infection. No severe infections occurred during treatment, with no cases requiring mechanical ventilation. Furthermore, there were no instances of belimumab discontinuation due to serious infections. Table 3 presents patients’ clinical characteristics at the last follow-up and the incidence of infection events throughout the follow-up period.

**Table 3:** Clinical characteristics of the study population at final follow-up and infectious events throughout follow-up.

Patient	Follow-up time (months)	Renal replacement therapy status at final follow-up	Extra-renal SLEDAI at final follow-up	Concomitant medications at final follow-up	GC dose at final follow-up^#^ (mg/day)	Infectious events	Severity of Infection (CTCAE Grade)	Time to infection from belimumab initiation (months)
1	19.3	MHD	0	none	0	Periodontal abscess	2	1
2	30.7	MHD	2 (C3↓)	HCQ	0	COVID-19 with bacterial superinfection	3	6.5
3	27.5	ESRD not yet on dialysis	2 (C3↓)	GC	2.5	None	Not applicable	Not applicable
4	42.0	MHD	2 (C3↓)	GC, HCQ	5	None	Not applicable	Not applicable
5	55.6	MHD	0	none	0	Pneumonia	3	2.5
6	21.0	MHD	0	GC, MMF	2.5	COVID-19 pneumonia	3	4
7	12.5	pre-Kidney transplantation	2 (C3↓)	GC, CTX	5	COVID-19, mild	2	2
8	15.5	MHD	4 (dsDNA+, C3↓)	GC, HCQ	10 / 7.5	Catheter-related bloodstream infection	3	15.5

GC, glucocorticoid; HCQ, hydroxychloroquine; RTX, rituximab; MMF, mycophenolate mofetil; CYC, cyclophosphamide. ^#^prednisone-equivalent.

## Discussion

Lupus nephritis (LN) remains one of the leading causes of end-stage renal disease (ESRD). In ESRD, the severe destruction of renal tissue and loss of kidney function are accompanied by diminished chemotactic and activation signals for inflammatory cells, resulting in reduced immune complex deposition and inflammatory infiltration. Notably, some studies have observed attenuated renal-specific indicators (e.g., proteinuria and hematuria) and improved systemic inflammatory markers (C-reactive protein, erythrocyte sedimentation rate) in ESRD patients, suggesting partial control of lupus activity (15). However, persistent extra-renal manifestations or heightened systemic disease activity may still occur in certain patients. Sustained extra-renal inflammation exacerbates the risks of vasculitis, accelerated atherosclerosis, and cardiovascular (CVD) events, ultimately contributing to poor clinical outcomes. These findings underscore the necessity for continued active management in ESRD patients with LN to achieve comprehensive disease control.

Current therapeutic management of this patient population remains challenging, largely due to the lack of evidence-based guidelines and standardized treatment protocols. Clinicians predominantly depend on the use of glucocorticoids and conventional immunosuppressants. Notably, hydroxychloroquine (HCQ)—a cornerstone in SLE management—is not routinely prescribed in ESRD patients because of heightened concerns about potential toxicity in this metabolically compromised population (16). Adverse effects of conventional immunosuppressive agents—including gastrointestinal complications, infections, and osteoporosis/pathological fractures—occur more frequently in ESRD patients. This heightened vulnerability is attributable to polypharmacy burden, gastrointestinal fragility, immune dysfunction, and the increased susceptibility to infections characteristic of this population (17). Notably, ESRD patients exhibit disturbances in mineral and bone metabolism, which further increases their risks of pathological fractures and CVD. The pharmacokinetic profiles of immunosuppressants such as cyclophosphamide and mycophenolate mofetil in ESRD patients exhibit marked unpredictability. This unpredictability is compounded by two critical challenges: (1) accumulation of nephrotoxic metabolites (e.g., cyclophosphamide) that may induce severe myelosuppression and (2) dialysis-mediated clearance of active drug components (e.g., mycophenolate mofetil) leading to subtherapeutic exposure levels. Therefore, this patient population exhibits reduced tolerance to conventional immunosuppressants and glucocorticoids, with an elevated risk of adverse effects.

Belimumab exerts its therapeutic effect by blocking B-lymphocyte stimulator (BLyS) activity, leading to B-cell depletion through exhaustion and apoptosis pathways, thereby suppressing autoantibody production and attenuating autoimmune responses. The drug’s efficacy and safety profile in systemic lupus erythematosus (SLE) management have been extensively validated across several clinical trials, particularly demonstrating advantages in three key aspects: disease activity control, reduction of flare frequency, and maintenance of clinical stability. The BLISS clinical trial series demonstrated significant clinical benefits of belimumab in reducing SLE disease activity (as assessed by SLEDAI scores) among patients. Patients in the belimumab treatment group demonstrated higher disease remission rates at 12 weeks and superior disease control compared to the placebo group (4). The BLISS-76 trial further confirmed belimumab’s long-term efficacy in SLE treatment, showing sustained disease activity control and significant clinical improvement in belimumab-treated patients at 52 weeks (5). These two trials demonstrated belimumab’s efficacy in reducing SLE disease activity and flare frequency, particularly showing superior therapeutic effects in patients with suboptimal responses to conventional therapies (e.g., glucocorticoids and immunosuppressants). The 2024 KDIGO Clinical Practice Guideline recommends belimumab as one of the adjunctive therapeutic options for patients with III/IV lupus nephritis to control disease activity and improve long-term prognosis. However, clinical data on the application of belimumab in SLE patients progressing to end-stage renal disease (ESRD) remain limited.

In this study, SLE patients who progressed to ESRD while maintaining belimumab therapy were prospectively followed and evaluated, with a mean follow-up duration of 23.5 months (range: 15–40 months). Results demonstrated that sustained belimumab treatment significantly reduced extra-renal SLE activity, particularly in controlling arthritis, cutaneous manifestations, reducing anti-dsDNA antibody titers, and restoring complement levels. Most patients achieved and maintained low SLEDAI scores post-treatment, consistent with findings from Liu D, Binda V, and Shota Ogura (10, 13, 14). Based on prior research, mechanistic exploration suggests downregulation of BLyS receptors on B cells in ESRD may paradoxically enhance BLyS overexpression. Additionally, deposition of anti-dsDNA antibodies in renal tubular injury constitutes a primary pathogenic mechanism of lupus nephritis, while studies demonstrate a positive correlation between BLyS levels and anti-dsDNA antibody titers. In summary, these findings confirm belimumab’s mechanism of action: inhibition of BLyS-mediated B-cell proliferation/differentiation, induction of B-cell exhaustion/apoptosis, and subsequent reduction of pathogenic autoantibody production. Notably, this therapeutic effect persists even in ESRD or dialysis-dependent LN patients, effectively mitigating the systemic lupus activity.

In contrast to the standard belimumab dosage (10 mg/kg) used in the BLISS trials, patients in this study received varying doses (4.3–10 mg/kg) at intervals ranging from every 4 weeks to 3 months, all achieving extra-renal disease control, glucocorticoid tapering, or sustained remission. Similarly, the case series by Shota Ogura and Snyder MS reported belimumab dose reduction due to infection-risk concerns, while maintaining therapeutic efficacy (12, 14). This phenomenon may be attributed to multiple factors in ESRD-SLE patients, including reduced immune activity, dialysis-mediated clearance of inflammatory mediators/immune complexes, prolonged belimumab half-life from altered drug metabolism/excretion, and interindividual variability in drug sensitivity/tolerance. These findings suggest that lower belimumab doses may suffice for disease control in ESRD-SLE patients, warranting personalized dose adjustments based on clinical status. First, it should be noted patients with ESRD and dialysis are inherently at high risk of infection. For example, in a randomized controlled trial (the HEMO study) of 1,846 long-term hemodialysis patients, the annual infection rate reached 35% over a mean follow-up of 2.8 years (18). Second, infection is the most common complication in LN patients (19, 20); therefore, the incidence of infection in patients with ESRD secondary to LN is expected to be higher, although exact research data are currently lacking. In our study, six episodes of infection occurred among eight patients during a mean follow-up of 28.01 ± 14.57 months, resulting in an overall infection incidence of 30.8 episodes per 100 patient-year, similar to the infection rate observed in the dialysis population in the HEMO study. Although the sample size is very limited, our data suggest that the use of belimumab did not increase the incidence of infection in this population.

Regarding combination therapy, this study demonstrated a significant glucocorticoid dose reduction in SLE patients who maintained belimumab treatment after progressing to ESRD. The mean daily prednisone dose decreased from 21.25 ± 9.64 mg/day at baseline to 3.91 ± 3.98 mg/day, with three patients achieving complete glucocorticoid discontinuation while maintaining disease remission. This substantially reduced glucocorticoid dependency and associated adverse effects, including infection risk, fluid retention, hyperglycemia, and osteoporosis. These findings align with outcomes from the BLISS-52 and BLISS-76 trials in non-ESRD populations.

Regarding treatment safety, among the eight patients, one discontinued belimumab due to leukopenia (<3.0 × 10^9^/L) and two episodes of pulmonary infections. One patient developed a periodontal abscess, four contracted COVID-19 (two with concurrent pneumonia, potentially related to the relaxation of pandemic control measures), and one experienced a tunnel infection involving a cuffed catheter (details in Table 3). No severe infections occurred, mechanical ventilation was not required, and no belimumab discontinuations were attributed to severe infections. Notably, baseline lymphocyte counts were below the normal ranges in several patients (Supplementary Tables 2, 3), suggesting preexisting immune dysfunction that may predispose to infections. However, frequent or severe infections were not observed during treatment. Furthermore, the review of published cases using belimumab in lupus nephritis with ESRD similarly reported no severe infections (Table 4). These findings indicate that belimumab demonstrates a favorable safety profile for ESRD-SLE patients.

**Table 4:** Summary of cases of lupus nephritis combined with end-stage renal disease treated with belimumab.

Published	Research	Results	Conclusion
Valentina Binda (13); 2020, Journal of Nephrology Italy	Seventeen patients with lupus nephritis were treated with belimumab for a period of 36 months to observe its effect on arthralgia, skin manifestations, proteinuria, and glucocorticoid reduction.	All patients experienced relief of arthralgia and skin manifestations, and glucocorticoids were successfully discontinued in six patients and reduced by approximately 40% in the others.	Belimumab has shown promising efficacy in patients with lupus nephritis, reducing disease activity and contributing to glucocorticoid tapering.
Shota Ogura (14); 2020. Blood purification Japan.	A 62-year-old woman patient diagnosed with SLE at the age of 12, who later developed end-stage renal disease (ESRD) and started dialysis for lupus nephritis, continued to be active.	The patient was treated with cyclophosphamide, mescaline, and prednisone, but side effects limited efficacy, and ultimately SLE activity declined rapidly after treatment with belimumab.	SLE in patients with end-stage renal disease is not significantly reduced after dialysis, and belimumab shows effectiveness in such patients.
Diankun Liu (10); 2022; The Lupus; China.	A retrospective analysis of seven SLE patients treated with belimumab was performed to observe changes in renal and immunologic markers.	All patients showed an improvement in immunologic markers and a significant decrease in disease activity, with five patients showing an increase in urine output and discontinuing dialysis, and only one patient continuing dialysis.	No serious adverse reactions were recorded, and there was only one case of pulmonary infection.
Chengning Zhang (11). 2022; Frontiers in immunology The China.	The efficacy and safety of four patients with severe active lupus nephritis treated with belimumab in combination with standard therapy while receiving renal replacement therapy are reported.	All four patients successfully discontinued RRT after belimumab in combination with standard therapy, with significant improvement in renal function, and no serious adverse events were reported.	Belimumab has shown favorable efficacy and safety in the treatment of patients with severely active LN.
Snyder MS (12); 2024; Journal of Clinical Rheumatology. America.	Medical record review of six patients with eGFR consistently below 30 mL/min / 1.73 m2 or requiring dialysis during treatment. The primary endpoint was to assess the safety of beribizumab by documenting the incidence of adverse events, including infections, laboratory abnormalities, malignancy, depression, suicide, and death. Secondary endpoints included SELENA-SLEDAI, steroid dose.	The majority of infection events were mild or moderate, with two cases of severe infection.	Belimumab may help to reduce SLE activity and steroid use. It is important to recognize the risk of infection, especially in patients on dialysis and concurrent immunosuppression.

## Conclusion

This study represents one of the few retrospective reports on belimumab use in lupus nephritis (LN) patients progressing to end-stage renal disease (ESRD), providing valuable insights into its application in this population. The findings of this study indicate that belimumab can significantly improve extra-renal disease activity, reduce glucocorticoid dosage, and demonstrate a favorable safety profile in ESRD-SLE patients. However, the limited sample size necessitates larger-scale prospective studies to establish the long-term safety and efficacy of belimumab in ESRD-LN populations.

## Data Availability

The original contributions presented in the study are included in the article/Supplementary material, further inquiries can be directed to the corresponding authors.
