# Supplementary material for: Efficacy of belimumab on extra-renal lupus in patients with lupus nephritis and end-stage renal disease
Source: Front Med (Lausanne). 2026 Jan 5;12:1717280. doi: 10.3389/fmed.2025.1717280 (PMC12812580; doi:10.3389/fmed.2025.1717280)
Supplement: Supplementary file 1 [file Data_Sheet_1.docx]

**Supplementary Table 1.** Changes in anti-dsDNA antibody and complement levels following belimumab therapy

| **Patient** | **Anti-dsDNA (IU/ml)** | | | | |  | **C3 (g/L)** | | | | |  | **C4 (g/L)** | | | | |
| --- | --- | --- | --- | --- | --- | --- | --- | --- | --- | --- | --- | --- | --- | --- | --- | --- | --- |
|  | **0m** | **3m** | **6m** | **12m** | **Final F/U** |  | **0m** | **3m** | **6m** | **12m** | **Final F/U** |  | **0m** | **3m** | **6m** | **12m** | **Final F/U** |
| 1 | 520 | 216 | 183.54 | 20.86 | 62.54 |  | 0.5 | 0.85 | 0.86 | 0.98 | 0.98 |  | 0.03 | 0.1 | 0.08 | 0.12 | 0.11 |
| 2 | 512.34 | 324.67 | 444.91 | 74.76 | NA |  | 0.77 | 0.75 | 0.95 | 0.73 | 0.88 |  | 0.19 | 0.18 | 0.24 | 0.21 | 0.21 |
| 3 | 48.44 | 20.95 | 19.27 | 5.75 | <10 |  | 0.71 | 0.72 | 0.63 | 0.46 | 0.56 |  | 0.14 | 0.13 | 0.14 | 0.14 | 0.13 |
| 4 | 19.76 | NA | 70.77 | negative | negative |  | 0.64 | 0.77 | 0.86 | 0.63 | 0.63 |  | 0.19 | 0.2 | 0.27 | 0.28 | 0.27 |
| 5 | 15.47 | 4.26 | NA | negative | negative |  | 0.85 | 0.99 | NA | 0.96 | 0.96 |  | 0.28 | 0.4 | NA | 0.38 | 0.38 |
| 6 | 1:10 (Titer) | NA | 1:10 (Titer) | negative | negative |  | 0.547 | NA | 1.29 | 0.9 | 0.9 |  | 0.23 | NA | 0.43 | 0.32 | 0.32 |
| 7 | 1:10 (Titer) | NA | negative | negative | negative |  | 0.61 | NA | 0.68 | 0.79 | 0.79 |  | 0.15 | NA | 0.21 | 0.21 | 0.21 |
| 8 | 150.8 | 197.77 | 145.23 | 116.69 | 180.9 |  | 0.53 | 0.49 | 0.71 | 0.60 | 0.66 |  | 0.02 | 0.06 | 0.14 | 0.09 | 0.07 |

Reference ranges are as follows. **Quantitative assays**: anti-dsDNA, 0-100 IU/mL; C3, 0.9-1.8 g/L; C4, 0.1-0.4 g/L. **Qualitative assays:** A negative result is considered normal for the anti-dsDNA and complement levels assays.

Abbreviations：m, month(s); Final F/U, final follow-up visit.NA, Not Available.

**Supplementary Table 2.** Changes in hematologic parameters after belimumab initiation

| **Patient** | **WBC (10^9^/L)** | | | | |  | **Lymphocytes (10^9^/L)** | | | | |  | **Hemoglobin (g/L)** | | | | |  | **Platelets (10^9^/L)** | | | | |
| --- | --- | --- | --- | --- | --- | --- | --- | --- | --- | --- | --- | --- | --- | --- | --- | --- | --- | --- | --- | --- | --- | --- | --- |
|  | **0m** | **3m** | **6m** | **12m** | **Final F/U** |  | **0m** | **3m** | **6m** | **12m** | **Final F/U** |  | **0m** | **3m** | **6m** | **12m** | **Final F/U** |  | **0m** | **3m** | **6m** | **12m** | **Final F/U** |
| 1 | 3.38 | 8.76 | 7.68 | 6.26 | 8.91 |  | 0.79 | 1.68 | 0.86 | 0.89 | 0.98 |  | 101 | 116 | 101 | 92 | 109 |  | 103 | 1569 | 137 | 144 | 157 |
| 2 | 5.8 | 5.85 | 4.56 | 4.63 | 6.71 |  | 0.46 | 0.54 | 0.4 | 0.94 | 1.72 |  | 114 | 114 | 91 | 123 | 116 |  | 164 | 199 | 182 | 230 | 271 |
| 3 | 4.93 | 5.97 | 4.05 | 4.1 | 4.04 |  | 1.21 | 0.51 | 1.17 | 0.98 | 1.09 |  | 107 | 115 | 111 | 109 | 111 |  | 164 | 151 | 158 | 152 | 167 |
| 4 | 6.38 | 7.93 | 5.17 | NA | 7.15 |  | 0.82 | 0.7 | 1.53 | NA | 0.73 |  | 94 | 89 | 70 | NA | 118 |  | 212 | 251 | 281 | NA | 248 |
| 5 | 9.46 | 4.85 | 6.58 | 13.55 | 5.55 |  | 1.49 | 1.22 | 1.1 | 1.6 | 1.3 |  | 104 | 97 | 125 | 120 | 109 |  | 193 | 146 | 166 | 132 | 203 |
| 6 | 3.9 | NA | 6.1 | 6 | 6 |  | 0.6 | NA | 0.6 | 1.2 | 1.2 |  | 58 | NA | 124 | 102 | 102 |  | 43 | NA | 75 | 165 | 165 |
| 7 | 2.37 | NA | 4.7 | NA | 4.7 |  | 1.32 | NA | 1.14 | NA | 1.1 |  | 118 | NA | 116 | NA | 116 |  | 87 | NA | 108 | 110 | 110 |
| 8 | 1.56 | 3.8 | 7.89 | 11.75 | 14.48 |  | 0.23 | 0.43 | 1.03 | 1.17 | 1.97 |  | 107 | 122 | 107 | 139 | 124 |  | 145 | 309 | 252 | 120 | 189 |

Abbreviations：m, month(s); Final F/U, final follow-up visit; NA, Not Available.

**Supplementary Table 3.** Longitudinal changes in serum IgG, CD19+ B-Cell count, and CD4+ T-Cell count after belimumab initiation

| **Patient** | **IgG (g/L)** | | | | |  | **CD19+ B-cells Count (cells/μL)** | | | | |  | **CD4+ T-cells Count (cells/μL)** | | | | |
| --- | --- | --- | --- | --- | --- | --- | --- | --- | --- | --- | --- | --- | --- | --- | --- | --- | --- |
|  | **0m** | **3m** | **6m** | **12m** | **Final F/U** |  | **0m** | **3m** | **6m** | **12m** | **Final F/U** |  | **0m** | **3m** | **6m** | **12m** | **Final F/U** |
| 1 | 9.75 | 8.25 | 7 | 6.34 | 7.47 |  | 39 | 42 | 24 | 33 | 21 |  | 173 | 525 | 365 | 333 | 326 |
| 2 | 10.5 | 9.89 | 9.1 | 7.66 | 10.68 |  | 49 | NA | NA | 14 | 7 |  | 222 | NA | NA | 486 | 217 |
| 3 | 13.53 | 12.1 | NA | 12.08 | 15.59 |  | 19 | 30 | 13 | 5 | NA |  | 701 | 799 | 602 | 665 | NA |
| 4 | 7.75 | 8.48 | 9.74 | NA | NA |  | 8 | NA | 5 | NA | 5 |  | 135 | NA | 178 | NA | 218 |
| 5 | 5.16 | 3.71 | 5.87 | 6.78 | 9.84 |  | 11 | 0 | 2 | 5 | 3 |  | 350 | 209 | 245 | 546 | 419 |
| 6 | 7.59 | NA | NA | NA | NA |  | 164.51 | NA | NA | NA | NA |  | 140.49 | NA | NA | NA | NA |
| 7 | NA | NA | NA | NA | NA |  | NA | NA | NA | NA | NA |  | NA | NA | NA | NA | NA |
| 8 | 8.09 | NA | NA | 6.64 | 7.3 |  | 1 | NA | NA | 29 | 25 |  | 444 | NA | NA | 298 | 532 |

Abbreviations：m, month(s); Final F/U, final follow-up visit; NA, Not Available.
